# The relationship between region of residence, socio-demographic factors, and healthcare utilization among Saudi citizens: insights from the 2013 Saudi Health Interview Survey

**DOI:** 10.3389/fmed.2023.1252340

**Published:** 2023-11-06

**Authors:** Motab Aljohani, Michael Donnelly, Ibrahim Al Sumaih, Ciaran O'Neill

**Affiliations:** ^1^Centre for Public Health, School of Medicine, Dentistry and Biomedical Sciences, Queen's University Belfast, Belfast, United Kingdom; ^2^Public Health Department, College of Health Science, Saudi Electronic, Riyadh, Saudi Arabia; ^3^Medical Supportive Services, King Fahad Hospital, Ministry of Health, Hofuf, Saudi Arabia

**Keywords:** healthcare access, primary care, healthcare utilization, health service use, healthcare determinants

## Abstract

**Background:**

There is a dearth of research on the nature and extent of variation in patterns of health service use in Saudi Arabia. This is an important gap in knowledge, given ongoing efforts to improve service provision and delivery. This study examined the relationship between the region of residence and socio-demographic factors and patterns of health service use in Saudi Arabia.

**Methods:**

Data were taken from the 2013 Saudi Health Interview Survey (SHIS), a national multistage survey of individuals aged 15 years and above in Saudi Arabia. Data included measures of service use, respondent health, socio-demographic characteristics, and region or area of residence. Descriptive statistics, Chi-square tests, and multivariable logistic regression analyses were used to describe the data and examine the likelihood of a respondent visiting a doctor or healthcare professional in the preceding 12 months. In addition, the analyses examined the role of health and socio-demographic characteristics within selected regions.

**Results:**

The increased likelihood of using health services in terms of visiting a doctor or healthcare professional was related to poor health status, being female, married, having a low income, and residing in particular regions. Respondents aged <65 and who lived furthest from service providers were less likely to visit a doctor or other health professionals (*p* < 0.01). Residents who lived in Riyadh, Al Medina, Baha, or Aseer demonstrated a higher likelihood of service utilization compared to respondents residing in other regions (*p* < 0.05). In sub-group analyses, there was variation between regions with respect to socio-demographic status and distance to service.

**Conclusion:**

Region of residence and income level, in particular, may help to explain the likelihood of primary care use in Saudi Arabia and the distinct patterns of service use in relation to regional and socio-demographic characteristics. The relationship between regional variation in service utilization and the socio-demographic characteristics of respondents may reflect differences with respect to population need, enabling, and predisposing factors as represented in Anderson's Behavioral Model (ABM) of health service use. The findings from this study underscore the importance of considering region or area of residence when seeking to understand the utilization of health services, particularly primary care services.

## Introduction

Primary care services are central to maintaining overall population health (1). They make an important contribution to cost containment (2) and to the promotion of equity across the healthcare system (3–5). Generally, primary care services are the first and often most frequent point of contact between the public and a healthcare system, and they occupy a pivotal role as gatekeepers to the hospital sector, ensuring appropriate referral to such services while offering continuity of care across the life course (6, 7). Therefore, it is important to improve our understanding of the factors that influence the utilization of such services, given their centrality to population health and the operation of the healthcare system. An examination of the patterns of service use provides an opportunity to ascertain the extent to which the current patterns of service use may deviate from the desired goals of policymakers as evidenced by unwarranted disparities in use or provide insights into the success of previous reforms intended to change patterns of service use. While valuable insights can be obtained from a comparative analysis of different primary care systems, as health systems differ across countries, there is a need to study primary care use within its particular policy, social, and cultural context.

In Saudi Arabia, primary care services are provided free at the point of use to all citizens through a system of primary care clinics operated across the country (8). The Ministry of Health (MOH) is the key provider and funder of such care, with 2,244 primary care centers in which doctors and other health professionals provide care to the population free of cost at the point of use (9). They provide access to a range of services, including prevention, management of chronic disease, and referral to other parts of the system (9). Other public agencies also operate primary centers for specific groups based on their employment, such as the National Guard or the Ministry of Defense. Further, private clinics also exist for those who wish to pay out of pocket or hold private insurance; however, the bulk of primary care remains provided through the MOH network (10). The primary care system provides national coverage, but variation in access exists between and within regions (9). In part, this is related to population density; urban centers and urbanized regions provide population concentrations that allow easier geographic access at a given level of supply per head of population. Between regions, however, levels of supply per head of population also vary. Thus, while, nationally, there are 2.2 centers per 10,000 persons in rural areas and 0.36 in urban areas, in urban areas of some regions, such as the Albaha region, the number of centers per 10,000 people is 1.1 while in urban areas such as Makkah, it is just 0.17 (9). Similar disparities exist between regions with respect to the number of centers in rural areas across regions and, no doubt, with respect to the size of centers. Such differences may have important implications for service use; those residing in areas with relatively low supply effectively face additional barriers to access.

Given the importance of primary care to health and healthcare, there is a disappointing paucity of research on primary care services in Saudi Arabia. The few studies that do exist, however, are often based on small sample sizes recruited from single regions. Al-Omar's research, which examined barriers to service use rather than use *per se* (11, 12), for example, used samples below 500 recruited from within one region. This was similarly the case with the study by Saeed and Mohamed (13). Similarly, the research by Mahfouz et al. (14) that examined utilization directly used small samples from within a single region, while a study by Alfaqeeh et al. (15) that examined differences in access to services between urban and rural areas was again concentrated within one region and excluded from among the factors examined the role of income in service use. Only one study we are aware of—by El Bcheraoui et al. (8)—has used national data to examine variations in utilization. While in their analysis, the authors account for respondent education, marital status, and aspects of health (history of diabetes and/or hypercholesterolemia), they did not account for the region or the respondent's income. More importantly, their focus is on the respondents' health, particularly diabetes and high cholesterol. The paucity of research on primary care is a consequence of limited value in understanding the use of primary care services more generally and the factors that influence it. As noted by Al Omar et al. (11), there is a dearth of studies based on Saudi data in this area, a dearth that appears not to have been addressed in the intervening period.

The Andersen Behavioral model (ABM) has been used to examine differences in the use of healthcare services in a variety of contexts (16–19). In brief, the model seeks to explain service use by reference to a range of observable characteristics possessed by the potential user. To illuminate the reasons underlying use, variables are grouped under three headings, including predisposing, enabling, and need factors. Predisposing factors are those that make a person more likely to use services, such as age, education, or cultural norms (20). These may result in a person being more aware of the existence or benefits of services, for example. Enabling factors include those related to the affordability of services, including the burden charges may present or that eligibility for support may offer. Barriers to service access related to travel times or the requirement to be accompanied by another person provide further examples of enabling factors (20). Need relates to both perceived and objectively measured needs covering, for example, self-assessed health as well as previously diagnosed conditions (20). Such factors may directly influence the perceived benefits of service use. Whether a particular socio-demographic characteristic is classed as an enabling or predisposing factor will depend on the particular context of a study. For example, where entitlement to publicly funded services is related to age, age might be thought of as an enabling rather than a predisposing factor. Our categorization follows that typically adopted in the literature (20, 21). Collectively, they may help guide policy in efforts to understand and address inequalities in service use and, consequently, health by identifying specific barriers that allow targeted intervention for particular groups, such as changes in employment rights, public funding, or tailored health promotion. In Saudi Arabia, as the health system is currently undergoing extensive reforms in line with the Kingdom's National Transformation Programme (22), an examination of the factors that influence the use of primary care is particularly apposite.

This study examines the relationship between regional and socio-demographic factors and patterns of health service use in Saudi Arabia. It extends the existing literature by explicitly incorporating individual income, region of residence, and a broader range of health characteristics using data collected from across Saudi Arabia.

## Materials and methods

### Data

The 2013 Saudi Health Interview Survey (SHIS), a national survey of individuals aged 15 and above in Saudi Arabia, was used to investigate the study's aims. The SHIS uses a multistage stratified probability sample to select study participants, ensuring representation from each region of the Kingdom of Saudi Arabia. The Kingdom was divided into clusters of households known as enumeration units, serving as primary sampling units (PSUs). The number of households in each PSU varied based on population, density, and geography. A probability proportional to size method was employed to randomly select PSUs from each of the 13 administrative regions. Within each PSU, 14 households were randomly chosen, totaling 12,000 households contacted. Out of these, 10,827 completed the survey. An additional 1,173 either partially completed or completed some sections but not the entire survey. Data included aspects of respondent health, socio-demographic characteristics, and region of residence. The survey explored the use of a range of services in sequence. Questions were grouped under separate headings, with individuals being asked if they used a clinic, hospital, or doctor/other health professional as options and asked after each option questions on the purpose of the visit and how recently it was made. We *assumed* questions related to doctor/other health professional for “illness, injury, immunization, other preventive service, and other” were likely to be primary care visits by virtue of the staff providing the service and the service being provided. The visits to a doctor or other healthcare professional constituted our measure of service use, though it is accepted that this was based on an assumption. No distinction was made between public and privately funded care. Descriptive statistics, Chi-square test, and multivariable logistic regression analyses were used to examine the relationship between the likelihood of visiting a doctor or other healthcare professional in the preceding 12 months. Respondent health and socio-demographic characteristics, including income and the region of residence, were included among the list of covariates. In subsequent analyses, the sample was categorized by region to examine the role of health and socio-demographic characteristics within selected regions. Analysis was confined to respondents for whom complete records were available. Further details about the survey are available on the SA Ministry of Health website (23).

### Analysis

The use of services was based on response to the question of whether the respondent had visited a doctor or health professional in the past 12 months (yes or no). Descriptive statistics (proportions together with their associated 95% confidence intervals) were used to describe the sample. Chi-square tests were used to compare groups differentiated with respect to morbidity, self-reported health, and income in terms of service use. Multivariable logistic regression analyses were undertaken in which the use of services in the preceding 12 months was specified as a function of a range of variables. Variable selection was determined by availability in the dataset and previous literature examining utilization with services (24). These were: self-reported health status—excellent, good, and very good was recorded as (good health status) where fair and poor was recoded as (poor health status) (poor vs. good) classified as need within the ABM; the number of illnesses the respondent reported having—it related to a list of potential conditions such as diabetes, asthma, blood pressure, and vision impairment; (morbidity), also classified as need within the ABM; age (65 and above vs. below 65), classified as a predisposing factor in the ABM; sex (male vs. female) classified as a predisposing factor in the ABM; educational status (literate or education qualifications vs. illiterate) classified as an enabling factor in the ABM; marital status (married vs. single, divorced, and widowed) classified as a predisposing factor in the ABM; obesity status (BMI ≥30) classified as a need factor in the ABM; income (categorized into eight groups, ranging from 3,000 Saudi Arabian Riyals (SAR) per month or less to 30,000 SAR or more, the lowest income group providing the base category) classified as an enabling factor in the ABM; travel distance to the service (categorized into five groups, with the nearest group being <1 km and the furthest group being 10 km or more, the nearest group providing the base category) classified as an enabling factor in the ABM; and region (all 13 regions of Saudi Arabia were specified, Baha providing the base category) classified as an enabling factor in our analysis. Logistic regressions were repeated for sub-groups based on region of origin. This was done to compare relationships between regions with respect to the role of covariates on the likelihood of service use, that is, to explore the possibility of distinct relationships for socio-demographic variables between regions. The sample analyzed was restricted to those who provided complete data—i.e., responses for all variables used in the analysis. For all analyses, significance was assessed at the *p* ≤ 0.05 level. As we were interested in differences between groups rather than reporting national estimates of use for groups, no attempt was made to weigh responses. All analyses were conducted in Stata version 16.0.

## Results

Table 1 presents the descriptive statistics of the sample. As can be seen, almost 75% of the sample visited a doctor or other health professional in the preceding 12 months, and over two-thirds traveled <5 km to see a doctor or healthcare professional, indicating the proximity of most individuals to a doctor's office. Approximately 56% of the sample were male, 69% were married, just over 8% were aged 65 and above, one-third were obese (BMI ≥30), 9% rated their health as poor, and almost 8.5% indicated one morbid condition.

**Table 1:** Descriptive statistics of the sample (*n* = 3,969).

**Variables**	**Frequency**	**Percentage %**	**Range (95% CI)**
Most recent doctor or healthcare professional visit in the past 12 months	2,964	74.7%	(73.3%−76%)
**Travel distance to health facility in km**			
Nearest radius (< 1 km)	849	21.4%	(20.1%−22.7%)
2nd (1–3 km)	1,106	27.9%	(26.5%−29.3%)
3rd (3–5 km)	723	18.2%	(17.%−19.4%)
4th (5–10 km)	658	16.6%	(15.4%−17.7%)
Furthest (>10 km)	633	15.9%	(14.8%−17.1%)
Travel distance in km median (IQR)		4.00	(2.00–8.00)
Males	2,208	55.6%	(54.1%−57.2%)
Education (literacy/education qualifications)	577	14.5%	(13.4%−15.6%)
Former/current smokers	797	20.1%	(18.8%−21.3%)
Age group (65 and above)	334	8.4%	(7.6%−9.3%)
Marital status (currently married)	2,748	69.2%	(67.8%−70.7%)
Self-reported health status (poor health)	366	9.2%	(8.3%−10.1%)
Obesity (BMI ≥30)	1,314	33.1%	(31.6%−34.6%)
Number of morbidities	3,580	0.90	(0.9–0.9)
**Income level**			
1st group (poorest)	621	15.6%	(14.5%−16.8%)
2nd	696	17.5%	(16.4%−18.7%)
3rd	665	16.8%	(15.6%−17.9%)
4th	773	19.5%	(18.2%−20.7%)
5th	669	16.9%	(15.7%−18%)
6th	314	7.9%	(7.1%−8.8%)
7th	122	3.1%	(2.5%−3.6%)
8th group (richest)	109	2.7%	(2.2%−3.3%)
**Region**			
Riyadh	428	10.8%	(9.8%−11.7%)
Jouf	166	4.2%	(3.6%−4.8%)
Western	614	15.5%	(14.3%−16.6%)
Almadina	223	5.6%	(4.9%−6.3%)
Qassem	138	3.5%	(2.9%−4.%)
East	300	7.6%	(6.7%−8.4%)
Aseer	306	7.7%	(6.9%−8.5%)
Tabouk	246	6.2%	(5.4%−6.9%)
Haiel	314	7.9%	(7.1%−8.8%)
Northern	327	8.2%	(7.4%−9.1%)
Jazan	276	7.0%	(6.2%−7.7%)
Najran	271	6.8%	(6.%−7.6%)
Baha	360	9.1%	(8.2%−10%)

Table 2 illustrates that as income increased, the percentage of those in poor health fell. This is unsurprising given the association normally found between good health and affluence. Tables 3, 4 provide the comparisons across groups with respect to morbidity or self-reported health, respectively. With respect to morbidity, this is defined in Table 3 as having at least one condition among those queried in the survey. While the figures for morbidity in each sub-group seem high, it should be remembered that here, the comparison refers to those who visited the doctor or other health professional. As can be seen, a higher proportion of respondents aged 65 and above reported that they had a morbid condition or disease, perhaps unsurprising given the known relationship between age and the accumulation of chronic conditions. Similarly, in Table 4, among those who visited the doctor or health professional, a higher percentage in the older age group than the younger age group described their health as poor. Similar results were observed for respondents who were illiterate compared to respondents with a higher level of education. Generally, the survey measures indicated that respondents who visited a doctor or health professional in the preceding 12 months had poorer self-reported health compared to respondents whose most recent visit was more than 12 months ago. 91.16% of those who had visited the doctor in the last 12 months (i.e., in 2013) had recorded a morbid condition. Among those whose last visit had been more than 12 months ago, the percentage with at least one morbid condition was 87.36%.

**Table 2:** Poor self-reported health status by income groups (*n* = 3,969).

**Variables**	**Income**	**Self-reported health**
Income	Group	Percentage (%)
	1st (poorest)	21.25%
	2nd	9.48%
	3rd	9.17%
	4th	5.69%
	5th	5.53%
	6th	5.73%
	7th	4.91%
	8th (richest)	1.83%

**Table 3:** Morbidity rate by socio-demographic and service utilization (*n* = 3,969).

**Variables**	**Morbidity**		
	**Percentage**	**Chi-square statistics**	***p*** **-value**
**Age**			
Under 65	89.43%	28.4461	^*^0.000
65 and above	98.50%		
**Sex**			
Female	90.91%	1.8316	0.176
Male	89.62%		
**Education**			
Illiteracy	96.18%	27.3850	^*^0.000
Literacy/have education qualification	89.18%		
**Marital status**			
Not married	82.88%	106.7766	^*^0.000
Currently married	93.44%		
**Doctor or health professional visit**			
Visited more than 12 months ago	87.36%	12.2424	^*^0.000
Visited in the past 12 months	91.16%		

The symbol “*” denotes statistical significance.

**Table 4:** Poor self-reported health by service utilization and socio-demographic (*n* = 3,969).

**Variables**	**Poor self-reported health**		
	**Percentage**	**Chi-square statistics**	***p*** **-value**
**Age**			
Under 65	6.54%	368.9626	^*^0.000
65 and above	38.32%		
**Sex**			
Female	11.52%	20.1097	^*^0.000
Male	7.38%		
**Education**			
Illiteracy	31.02%	383.3304	^*^0.000
Literacy/have education qualification	5.51%		
**Marital status**			
Not married	10.07%	1.5301	0.2162
Currently married	8.84%		
**Doctor or health professional visit**			
Visited more than 12 months ago	4.87%	30.3623	^*^0.000
Visited in the past 12 months	10.69%		

The symbol “*” denotes statistical significance.

Table 5 presents the results of the examination of differences in the use of doctors or other health professional services in the past 12 months. Consistent with expectations, a higher proportion of those who were aged 65 or above had morbidity, and those who reported health as poor were more likely to have visited the doctor within the last 12 months than their comparators. Women were significantly more likely to have used services in the past 12 months than men. Interestingly, while married respondents were more likely to report having morbidity (Table 3), they were not more likely to report poor health (Table 4) but were more likely to have reported use of a primary care center in the past 12 months. This is suggestive of married individuals perhaps having a lower threshold in self-reported health for triggering a visit to the doctor.

**Table 5:** Comparison of service utilization by socio-demographic morbidity and health status (*n* = 3,969).

**Variables**	**Doctor or health professional visit**		
	**Percentage**	**Chi-square statistics**	***p*** **-value**
**Age**			
Under 65	73.53%	29.8792	^*^0.000
65 and above	87.12%		
**Sex**			
Female	76.88%	8.1714	0.004
Male	72.91%		
**Education**			
Illiteracy	82.14%	19.9248	^*^0.000
Literacy/have education qualification	73.40%		
**Marital status**			
Not married	71.82%	7.5878	0.006
Currently married	75.94%		
**Morbidity**			
No	67.35%	12.2424	^*^0.000
Yes	75.47%		
**Self-reported health**			
Good	73.46%	30.3623	^*^0.000
Poor	86.61%		

The symbol “*” denotes statistical significance.

Table 6 presents the results of a logistic regression analysis of the relationship between a self-reported visit to a doctor or other health professional in the past 12 months and a range of variables. Distance to the facility where the service was provided was not significantly related to the use of services in the past 12 months, perhaps unsurprising given the relatively short distances respondents typically had to travel. Respondents who were men were almost 30% points less likely to have visited in the past 12 months, as indicated by the adjusted odds ratio. Those who were aged 65 and above, married, reported poorer health, or reported having an ill-health condition or disease were also more likely to have visited than their respective comparators. These results are broadly consistent with expectations of men typically being less likely to use services those who are older, sicker, and married being more likely to use services (8, 11, 15). Notably, the highest income group was almost 70% less likely to have visited in the past 12 months than the poorest group of respondents. Further, the place of residence/region appeared to be statistically significant—respondents who were residents in Qassem, Northern, and Najran, for example, were almost 60% less likely to have visited a doctor or other health professional in the past 12 months compared to residents from Baha.

**Table 6:** Logistic regression analysis of the relationship between visiting (or not) a doctor or health professional in the past 12 months and socio-demographic variables (*n* = 3,969).

	**OR**	**95% CI**	***p*-value**
**Travel distance to health facility**			
Nearest radius (< 1 km)	ref		
2nd (1–3 km)	1.00	(0.81–1.24)	0.97
3rd (3–5 km)	0.91	(0.72–1.16)	0.47
4th (5–10 km)	0.83	(0.65–1.07)	0.15
Furthest (>10 km)	0.86	(0.67–1.1)	0.24
**Travel distance in km median (IQR)**			
Sex (being male)	0.81	(0.69–0.96)	0.017
Education (literacy/education qualifications)	1.05	(0.8–1.37)	0.74
Former/current smokers	0.91	(0.74–1.1)	0.33
Age group (65 and above)	1.89	(1.31–2.72)	0.001
Marital status (currently married)	1.28	(1.08–1.51)	0.003
Self-reported health (poor rated)	1.77	(1.28–2.44)	0.001
Obesity (BMI ≥30)	1.04	(0.88–1.23)	0.62
Morbidity	1.32	(1.04–1.69)	0.03
**Income status**			
1st group (poorest)	ref		
2nd	0.94	(0.72–1.24)	0.67
3rd	0.78	(0.59–1.02)	0.07
4th	0.92	(0.7–1.21)	0.55
5th	0.83	(0.62–1.1)	0.19
6th	1.12	(0.78–1.61)	0.55
7th	1.00	(0.62–1.61)	0.99
8th group (richest)	0.32	(0.2–0.51)	< 0.001
**Regions**			
Riyadh	1.00	(0.68–1.46)	0.98
Jouf	0.51	(0.32–0.79)	0.003
Western	0.54	(0.38–0.76)	< 0.001
Almadina	0.89	(0.56–1.39)	0.6
Qassem	0.43	(0.27–0.69)	0.001
East	0.50	(0.34–0.74)	< 0.001
Aseer	0.83	(0.55–1.26)	0.39
Tabouk	0.50	(0.33–0.75)	0.001
Haiel	0.65	(0.44–0.96)	0.03
Northern	0.45	(0.31–0.67)	< 0.001
Jazan	0.64	(0.43–0.96)	0.03
Najran	0.43	(0.29–0.64)	< 0.001
Baha	ref		

Tables 7A–C present the results of the logistic regression analyses that examined the likelihood of service use with respect to specific regions. The variables related to service use appeared to differ between regions. For example, distance was significant in the Eastern region but not in Riyadh or the Northern regions; age and self-reported health status were significant in Riyadh, but neither variable was significant in the Eastern or Northern regions. Similarly, while only the highest income category was significant in Eastern and Riyadh regions, it was the middle-income categories that were significantly lower in the Northern region, and those in the highest category were not significantly different from the lowest.

**Table 7A:** Logistic regression of visiting a doctor or healthcare professional in Riyadh region (*n* = 405).

	**OR**	**95% CI**	***p*-value**
**Travel distance to health facility**			
Nearest radius (< 1 km)	ref		
2nd (1–3 km)	0.39	(0.15–1.05)	0.06
3rd (3–5 km)	0.92	(0.33–2.54)	0.87
4th (5–10 km)	1.09	(0.39–3.01)	0.87
Furthest (>10 km)	1.25	(0.39–4.08)	0.71
Sex (being male)	0.55	(0.29–1.03)	0.062
Education (literacy/education qualifications)	1.71	(0.19–15.43)	0.63
Former/current smokers	1.30	(0.67–2.51)	0.44
Age group (65 and above)	1.00	(0.0−0.0)	< 0.001
Marital status (currently married)	1.45	(0.81–2.61)	0.211
Self-reported health (poor rated)	8.30	(1.09–63.33)	0.04
Obesity (BMI ≥30)	0.61	(0.34–1.1)	0.1
Morbidity	1.11	(0.49–2.5)	0.8
**Income status**			
1st group (poorest)	ref		
2nd	1.55	(0.34–7.12)	0.57
3rd	1.45	(0.31–6.75)	0.63
4th	1.75	(0.38–8.08)	0.47
5th	1.01	(0.23–4.44)	0.99
6th	2.71	(0.53–13.76)	0.23
7th	5.22	(0.44–62.33)	0.19
8th group (richest)	0.14	(0.02–0.97)	0.05

**Table 7B:** Logistic regression of visiting a doctor or healthcare professional in Eastern region (*n* = 300).

	**OR**	**95% CI**	***p*-value**
**Travel distance to health facility**			
Nearest radius (< 1 km)	ref		
2nd (1–3 km)	1.75	(0.86–3.56)	0.12
3rd (3–5 km)	2.37	(0.87–6.46)	0.09
4th (5–10 km)	3.77	(1.56–9.11)	0.000
Furthest (>10 km)	1.90	(0.81–4.47)	0.14
Sex (being male)	0.54	(0.29–1.03)	0.062
Education (literacy/education qualifications)	0.93	(0.23–3.81)	0.92
Former/current smokers	1.11	(0.55–2.26)	0.77
Age group (65 and above)	1.11	(0.23–5.42)	0.9
Marital status (currently married)	1.69	(0.92–3.11)	0.089
Self-reported health (poor rated)	2.28	(0.43–12.17)	0.34
Obesity (BMI ≥30)	1.08	(0.58–2.01)	0.81
Morbidity	1.53	(0.56–4.2)	0.41
**Income status**			
1st group (poorest)	ref		
2nd	2.64	(0.85–8.16)	0.09
3rd	0.65	(0.22–1.9)	0.43
4th	1.39	(0.47–4.1)	0.56
5th	1.28	(0.42–3.89)	0.66
6th	0.84	(0.23–3.06)	0.79
7th	1.27	(0.27–5.97)	0.76
8th group (richest)	0.16	(0.03–0.93)	0.04

**Table 7C:** Logistic regression of visiting a doctor or healthcare professional in Northern region (*n* = 327).

	**OR**	**95% CI**	***p*-value**
**Travel distance to health facility**			
Nearest radius (< 1 km)	ref		
2nd (1–3 km)	0.88	(0.49–1.55)	0.65
3rd (3–5 km)	0.75	(0.36–1.58)	0.46
4th (5–10 km)	3.00	(0.3–29.49)	0.35
Furthest (>10 km)	1.00	(0.28–3.57)	1.
Sex (being male)	0.81	(0.44–1.5)	0.506
Education (literacy/education qualifications)	0.84	(0.33–2.15)	0.71
Former/current smokers	0.70	(0.36–1.35)	0.29
Age group (65 and above)	1.65	(0.41–6.68)	0.48
Marital status (currently married)	1.64	(0.91–2.93)	0.099
Self-reported health (poor rated)	2.27	(0.72–7.19)	0.16
Obesity (BMI ≥30)	1.10	(0.62–1.94)	0.75
Morbidity	1.30	(0.61–2.78)	0.5
**Income status**			
1st group (poorest)	ref		
2nd	0.57	(0.22–1.46)	0.24
3rd	0.35	(0.14–0.88)	0.03
4th	0.37	(0.14–0.97)	0.04
5th	0.35	(0.13–0.97)	0.04
6th	0.24	(0.07–0.87)	0.03
7th	0.24	(0.06–1.06)	0.06
8th group (richest)	0.09	(0.01–1.16)	0.07

## Discussion

In most healthcare systems, primary care is the first and most frequent point of contact between the public and the health service. It occupies a pivotal role in referral to secondary services and is central to the efficient and equitable operation of the entire service. There is a paucity of research about variations in the use of health services (11) in Saudi Arabia, with even fewer studies that are population-based. The study by El Bcheraoui et al. (8) is a notable exception but is also limited in terms of its generalisability by virtue of its focus on the diagnosis and treatment of a specific patient sub-group (specifically those with hypertension, diabetes, and hypercholesterolemia) as opposed to utilization by the public generally. The few studies that have examined the use tend to have small samples and, as noted, do not take into account income or region—factors that are known to affect utilization in other healthcare systems (25). Studies have demonstrated the existence of variation in access to services (9), though, within one region, access was not found to be influenced by rural or urban status (15). Other studies have also demonstrated the importance of income, suggesting these factors should be featured in an analysis relating to utilization. Our study is the first one that we are aware of that has used national data to look at utilization beyond a specific group of patients, control for income, and compare utilization across regions and differences related to socioeconomic status between regions (9). As such, it makes an important contribution to the literature.

The use of ABM allows us to interpret results in terms of the influence of specific variables as contributing to need, predisposing, and enabling factors and to relate our findings to those of others. Our findings that older and married respondents and those with poorer health are more likely to use services, as shown in Table 6, are consistent with previous studies in other health system contexts (6). That income is largely unrelated to service use again finds echoes in studies by van Doorslaer et al. (5), though this does vary between contexts dependent on the incentives facing practitioners (26) and users of services (27). In this study, only the highest level of income was significant within the main analysis presented in Table 6, suggesting its role may be confined to a relatively small group. Given access to publicly funded services in Saudi Arabia is free at the point of use and given the specification of use adopted in this study (any use within the previous 12 months), it seems unlikely that the issue of service affordability explains the observed finding. It seems more likely that those in the highest income group are healthier, something supported by the results reported in Table 2. This would see income act as a surrogate for need rather than as an enabling factor. An alternative explanation is that the opportunity of time to this group is higher, which makes them less likely to visit care providers, other things being equal, an argument that has been made by McGregor et al. (28). Income that is among this group raises the cost of visiting the doctor and serves to deter use. If this is the case, it could be such individuals may require more flexible arrangements to support access to care, such as out-of-hours access or online consultations. For those outside this highest income group, the results suggest that there exists a considerable degree of equity in the use of services with respect to income, something that may reassure those responsible for funding services and that stands in contrast to studies of systems where a greater role exists for private care such as in Ireland (29).

Within the context of the ABM model, it is easy to conceptualize the increased likelihood of use related to health as related to needs. Thus, older individuals and those who report poor health or have been diagnosed with morbidity would be likely to exhibit a greater need for medical care. Our finding echoes those from other studies (28, 30). With respect to marital status, the likelihood that partners may encourage a respondent to increase use, as noted, was viewed as a predisposing factor. As married couples share the responsibility for raising a family, they are dependent on their partners to meet those responsibilities. It is perfectly understandable, therefore, that partners would be more likely to ensure both partners invest in their health to meet those responsibilities by encouraging each other to visit the doctor. That distance (where proximity could be viewed as an enabling factor) is unrelated to service use could reflect the specification of the dependent variable in our analysis (use in the past 12 months). Here, the distances over which a person may have to travel may be unlikely to be sufficient to affect the likelihood of use over the course of a year but could readily affect the frequency of use conditional on any use. This is an area that warrants further investigation.

That place of residence/region is significant (see Table 6) and that distinct relationships exist between socio-demographic variables and the likelihood of service use across regions (see Table 7) underscores the importance of the inclusion of place of residence in the analyses. Clearly, not only do differences exist between regions, but also in the relationships between use and individual characteristics across regions. In our analysis, we have interpreted region as an enabling factor—likely capturing differences in the number of doctors per head per region as well as relative rurality, that is, how easily they can be reached. This has potentially important policy implications. Lower use in Jouf, Western, or Qassem regions compared to Baha may, for example, be grounded in a mismatch between supply and population needs in these regions and may require investment in these regions to address inequalities in access. That is, it may, consistent with the analysis of Saffer et al. (9), be indicative of supply-side issues that could be readily addressed by increasing supply. Equally, though it may relate to a cultural mismatch in how services are provided in these regions, it is not the level of service but the manner of provision that affects use. This may require a change to how services are provided as opposed to the level of provision consistent with the arguments of Al-Omar et al. (11) or Al-Shahri (31). This is an area that could benefit from further investigation.

The role of sex, seen within the ABM as a predisposing factor, warrants comment. In Saudi Arabia, it remains customary for females to be accompanied by a male family member when visiting health facilities to seek medical care. Males in our sample were ~20% less likely to have visited a doctor or other health professional in the past 12 months than females (Table 6), controlling for other variables. While males in our sample were healthier than females (Tables 3, 4), and there may be a degree of unobserved heterogeneity related to health in the function, it remains that health status is among the variables controlled for in our regression in Table 6. This suggests that males do not view accompaniment as attendance at the doctor or health professional—the survey question on use has, in other words, not been interpreted as extending to accompaniment. By extension, it suggests that visits to a health facility as a chaperone by men offer a potentially missed opportunity for them to avail of health services at the facility, for example, preventive services. If this is the case, availing of the opportunity to offer services to men might bring direct benefits to them in terms of advice or early detection and better management of health issues. Importantly, however, it may also bring indirect benefits to women by providing an additional incentive for men to accompany female family members on health facility visits, thus reducing barriers they might otherwise encounter. Encouraging such measures might not only help to reduce the gap in life expectancy between the sexes in Saudi Arabia but also improve the life expectancy of females in Saudi Arabia, which lags behind that of females in many other Gulf states (32). Again, this is an area that warrants further investigation.

## Strengths and limitations

The study has a number of strengths. That the sample covers the entire country allows for an examination of regional effects, on which there is currently a dearth of studies in the Kingdom of Saudi Arabia. The sample size is large, significantly larger than several previous studies (11–13, 33). The survey also allowed for a reasonably detailed characterization of the respondents in terms of health and socio-demographic characteristics. There are, however, also a number of limitations to the study. First, the dependent variable, use of service in the previous 12 months rather than the number of times the service was used, provides a rather blunt measure of service use that does not allow us to examine variations in visit frequency, which would have been instructive. Second, the measure does not allow us to distinguish between publicly funded and privately funded services and examine differences in patterns of service use between them. This would have been interesting and may, for example, have sharpened the analysis of effects related to public use and enabling factors. Another notable limitation is the age of the data, sourced from 2013; more recent national data might have been more informative and relevant. Third, the survey question does not explicitly identify primary care as the type of service used. We have inferred this based on the context of the question within the survey and its link to the question that follows immediately afterward on conditions one would expect to see managed in primary care. This remains an assumption, however, and some caution is, in consequence, warranted with our interpretation of findings.

## Conclusion

Our study sheds light on the need for more research into healthcare utilization in the Kingdom of Saudi Arabia, particularly given the limited literature in this area. Our findings emphasize the importance of considering regional influences when interpreting the role of specific variables in healthcare utilization. We suggest that future studies should pay careful attention to regional differences when examining the role of specific variables and explore the potential impact of cultural and social factors on healthcare utilization. Overall, our study highlights the need for more research in order to enhance the understanding of variations in service use and increase the evidence-based redesign of services that will improve health outcomes and reduce healthcare disparities in the Kingdom of Saudi Arabia.

## Data availability statement

The data analyzed in this study is subject to the following licenses/restrictions. Data sharing is restricted as the data belongs to Ministry of Health in Saudi Arabia and only is available from corresponding author upon reasonable request. Requests to access these datasets should be directed to Maljohani01@qub.ac.uk.

## Author contributions

MA, MD, and CO'N conceived and drafted the study. MA and CO'N designed the research. MA analyzed the data. MA, MD, CO'N, and IA contributed to the drafting of the paper. All authors approved the final draft of the manuscript.
